# Identification of the flotillin-1/2 heterocomplex as a target of autoantibodies in *bona fide* multiple sclerosis

**DOI:** 10.1186/s12974-017-0900-z

**Published:** 2017-06-23

**Authors:** S. Hahn, G. Trendelenburg, M. Scharf, Y. Denno, S. Brakopp, B. Teegen, C. Probst, K. P. Wandinger, M. Buttmann, A. Haarmann, F. Szabados, M. vom Dahl, T. Kümpfel, P. Eichhorn, H. Gold, F. Paul, S. Jarius, N. Melzer, W. Stöcker, L. Komorowski

**Affiliations:** 1 Institute of Experimental Immunology, Euroimmun AG, Seekamp 31, 23560 Lübeck, Germany; 2 Department of Neurology, University Medical Center Göttingen, Göttingen, Germany; 3 Clinical Immunological Laboratory Prof. Dr. med Stöcker, Lübeck, Germany; 4 Department of Neurology, University Medical Center Schleswig Holstein (UKSH), Lübeck, Germany; 5 Department of Neurology, University of Würzburg, Würzburg, Germany; 6 Department of Neurology, Caritas Hospital, Bad Mergentheim, Germany; 7 Medical Laboratory Osnabrück, Georgsmarienhütte, Germany; 8 Department of Neurology, Ammerland Klinik, Westerstede, Germany; 9 Institute of Clinical Neuroimmunology, Ludwig Maximilian University, Munich, Germany; 10 Institute of Clinical Chemistry, Ludwig Maximilian University, Munich, Germany; 11 Department of Neurology, Klinikum am Gesundbrunnen, Heilbronn, Germany; 12 NeuroCure Clinical Research Center and Clinical and Experimental Multiple Sclerosis Research Center, Department of Neurology, Charité Universitätsmedizin, Berlin, Germany; 13 Experimental and Clinical Research Center, Max Delbrück Center for Molecular Medicine and Charité Universitätsmedizin, Berlin, Germany; 14 Department of Neurology, University of Heidelberg, Heidelberg, Germany; 15 Department of Neurology, University of Münster, Münster, Germany

**Keywords:** Autoantibodies, Flotillin, Reggie, Histo-immunoprecipitation, Multiple sclerosis, Optic neuritis

## Abstract

**Background:**

Autoantibodies, in particular those against aquaporin-4 and myelin-oligodendrocyte glycoprotein (MOG), aid as biomarkers in the differential diagnosis of demyelination. Here, we report on discovery of autoantibodies against flotillin in patients with multiple sclerosis (MS).

**Methods:**

The target antigen was identified by histo-immunoprecipitation using the patients’ sera and cryosections of rat or pig cerebellum combined with mass spectrometrical analysis. Correct identification was ascertained by indirect immunofluorescence and neutralization tests using the target antigens recombinantly expressed in HEK293 cells.

**Results:**

Serum and CSF of the index patient produced a fine-granular IgG indirect immunofluorescence staining of the hippocampal and cerebellar molecular layers. Flotillin-1 and flotillin-2 were identified as target autoantigens. They also reacted with recombinant human flotillin-1/2 co-expressed in HEK293 cells, but not with the individual flotillins in fixed- and live-cell assays. Moreover, neutralization using flotillin-1/2, but not the single flotillins, abolished the tissue reactivity of patient serum. Screening of 521 patients, for whom anti-aquaporin-4 testing was requested and negative, revealed 8 additional patients with anti-flotillin-1/2 autoantibodies. All eight were negative for anti-MOG. Six patients *ex post* fulfilled the revised McDonald criteria for MS. Vice versa, screening of 538 MS sera revealed anti-flotillin-1/2 autoantibodies in eight patients. The autoantibodies were not found in a cohort of 67 patients with other neural autoantibody-associated syndromes and in 444 healthy blood donors.

**Conclusions:**

Autoantibodies against the flotillin-1/2 heterocomplex, a peripheral membrane protein that is involved in axon outgrowth and regeneration of the optic nerve, are present in 1–2% of patients with *bona fide* MS.

**Electronic supplementary material:**

The online version of this article (doi:10.1186/s12974-017-0900-z) contains supplementary material, which is available to authorized users.

## Background

Several immune-mediated inflammatory central nervous system (CNS) disorders are characterized by the presence of autoantibodies against components of neural tissues [1]. Moreover, a significant number of autoantibodies with pathogenic potential, e.g., directed against aquaporin-4, have been described [2–7]. Autoantibodies against aquaporin-4 (AQP4) cause inflammation in the CNS and can be regarded as pathognomonic for neuromyelitis optica spectrum disorder (NMOSD) [2, 8–10]. Lead symptoms are uni- or bilateral optic neuritis (ON) together with longitudinal extensive transverse myelitis [11]. ON and transverse myelitis are also frequent initial symptoms in multiple sclerosis (MS). Most MS patients develop ON at least once during the disease course [12], and conversely, both children and adults with ON have an elevated risk of developing MS [13, 14]. Generally, neuroimaging and laboratory testing help to diagnose and differentiate MS, NMOSD, and other forms of central demyelination [10]. However, intrathecal immunoglobulin synthesis and oligoclonal bands in the CSF, frequently detected in patients with MS, are not disease-specific. Searching for anti-AQP4 has therefore become an important step in the diagnostic work-up of patients under suspicion of having a central demyelinating disease. Broad screening for multiple autoantibodies is justified at this stage because they can be linked to phenotypically diffuse neurological syndromes, especially in early disease stages. Conclusively, *ex ante* unexpected findings are frequent in the serological laboratory.

Here, we report on autoantibodies against the flotillin-1/2 complex detected in patients referred for anti-AQP4 testing, most of whom turned out to suffer from *bona fide* multiple sclerosis presenting with ON. Flotillin-1 and flotillin-2 (reggie-2 and reggie-1) are homologous scaffolding proteins of lipid raft microdomains with a molecular mass of 47 kDa [15, 16]. They are highly conserved and ubiquitously expressed [17]. Membrane association of flotillins depends amongst other factors on palmitoylation at Cys34 of flotillin-1 [18, 19] and Cys4, Cys19, and Cys20 of flotillin-2 [20, 21]. The flotillin proteins form hetero-oligomeric complexes and were originally named reggies due to their upregulation in regenerating axons of goldfish retinal ganglion cells after lesion of the optic nerve [22]. Furthermore, they play an important role in cell proliferation, tumor progression, neurodegenerative diseases, and signaling in T cells. Flotillin microdomains form flotillin cap at one pole of T cells regulating recruitment of signaling molecules to the cap which is important for T cell activation [15].

## Methods

Patients were analyzed as part of their diagnostic work-up based on the suspicion of a neuroinflammatory disease by a broad screening for autoantibodies. Serum and CSF samples were initially investigated on the assumption of an autoimmune disorder of the CNS in the accredited Clinical Immunological Laboratory in Lübeck by indirect immunofluorescence assay (IFA) using a combination of brain tissue cryosections and HEK293 cells expressing established neural autoantigens between May 2012 and September 2015.

The six patients revealed in the initial screening were diagnosed and treated at the Departments of Neurology of the contributing Medical Centers and Hospitals. All patients were referred for serology as part of their diagnostic work-up based on the clinical differential diagnosis between MS and NMOSD mostly due to clinical presentation with prominent ON by a broad screening for autoantibodies. The index patient (P1) gave written informed consent for using her serum and CSF samples for autoantigen identification. All six patients for whom detailed data are reported consented to the serological analysis of their samples and the subsequent evaluation of their medical records.

Control collectives included 538 serum samples from random patients with pre-diagnosed MS, 444 healthy blood donors, and 67 patients with defined autoantibody-associated neurological syndromes (5x anti-NMDAR, 5x anti-Hu, 2x anti-Hu/anti-Ri, 3x anti-Yo, 2x anti-Yo/anti-Ri, 3x anti-Ri, 38x anti-AQP4, 5x anti-LGI1, 4x anti-CASPR2).

Reagents were obtained from Euroimmun (Lübeck, Germany) or Merck (Darmstadt, Germany) or Sigma-Aldrich (Heidelberg, Germany) if not specified otherwise.

### Histo-immunoprecipitation and identification of the antigen

The cerebellum from rat or pig was dissected and shock-frozen in −160 °C isopentane. The tissue was then cryosected (4 μm) with a SM2000R microtome (Leica Microsystems, Nussloch, Germany), placed on glass slides, dried, and stored at −196 °C. For histo-immunoprecipitation (HIP), the slides were incubated with the patient’s serum (diluted 1:100) at 4 °C for 3 h followed by three washing steps with PBS, 0.2% (*w*/*v*) Tween-20 (IFA buffer). The tissue was then extracted with solubilization buffer (100 mmol/l tris-HCl pH 7.4, 150 mmol/l sodium chloride, 2.5 mmol/l EDTA, 0.5% (*w*/*v*) deoxycholate, 1% (*w*/*v*) Triton X-100 containing protease inhibitors) at 4 °C for 1 h. The resulting suspension was homogenized and centrifuged at 16,000×*g* at 4 °C for 15 min. Immunocomplexes were precipitated from the clear supernatant with Protein G Dynabeads (ThermoFisher Scientific, Dreieich, Germany) at 4 °C overnight, washed three times with solubilization buffer, and eluted with NuPAGE LDS Sample Buffer (Thermo Fisher Scientific, Dreieich, Germany) at 70 °C for 10 min. The eluates were analyzed by SDS-PAGE and mass spectrometry or Western blot.

### Indirect immunofluorescence assay

Slides with biochip mosaic including brain tissue cryosections (rat hippocampus and rat, pig, and primate cerebellum) and HEK293 cells expressing wild-type and mutated flotillin-1 and flotillin-2 as well as 30 recombinant brain antigens were used for IFA. Each mosaic was incubated with 30 μl of sample diluted in PBS, 0.2% Tween-20 (IFA buffer) at room temperature for 30 min; flushed with IFA buffer; and immersed in IFA buffer for 5 min. Subsequently, polyclonal goat anti-human pan-IgG (Euroimmun, Lübeck, Germany) or monoclonal murine anti-human IgG1, IgG2, IgG3, or IgG4 (Sigma-Aldrich, Heidelberg, Germany), each labeled with fluorescein isothiocyanate (FITC) or Alexa Fluor 488, were incubated at room temperature for 30 min. The slides were then washed again; embedded in PBS-buffered, DABCO-containing glycerol (approximately 10 μl per mosaic); and examined by two independent observers using an EUROStar microscope (Euroimmun, Lübeck, Germany) and a laser scanning microscope (LSM700, Zeiss, Jena, Germany). Positive and negative controls were included. Samples were categorized based on tissue patterns and fluorescence intensity of transfected cells in direct comparison with non-transfected cells and control samples. Endpoint titers refer to the highest dilution showing visible fluorescence. Live-cell IFA with primary hippocampal neurons or transfected HEK293 cells was conducted as described by Dalmau et al. [3].

Polyclonal rabbit antibodies against flotillin-1 and flotillin-2 (both Sigma-Aldrich, Heidelberg, Germany; dilution 1:250), respectively, were used in some experiments in the first step followed by incubation with anti-rabbit IgG-Cy3 (Jackson Research, Suffolk, UK). Cell nuclei were visualized by DNA staining with TO-PRO-3 iodide (ThermoFisher Scientific, Dreieich, Germany; dilution 1:2000). Recombinant antigens were mixed with diluted serum sample 1 h at room temperature prior to IFA as described in Stöcker et al. for neutralization experiments [23].

See Additional file 1 for further details.

## Results

### Characterization of the patient’s autoantibodies

**Fig. 1:**
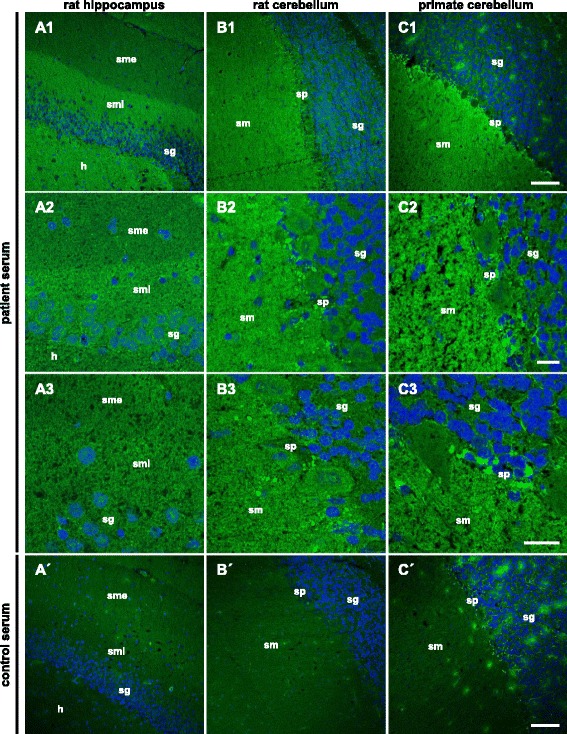
Immunofluorescence staining of central nervous tissues. Cryosections of rat hippocampus (**A**) and cerebellum (**B**) as well as primate cerebellum (**C**) were incubated with patient serum (**A**–**C**) or with control serum (*A′–C′*) (*A*–*C1*, *A′–C′* 1:100, *A*–*C2*, *C3* 1:50) in the first step and with Alexa Fluor 488-labeled goat anti-human IgG in the second step (*green*). Nuclei were counterstained by incubation with TO-PRO-3 iodide (*blue*). A fine-granular staining of the stratum moleculare (*sm*) was obtained. On the hippocampus, the sm internum was stained more intensely than the sm externum. *Scale bar*: 100 μm (*A*–*C1*, *A′–C′*), 20 μm (*A*–*C2, C3*). *h* hilus, *sm* stratum moleculare, *smi* stratum moleculare internum, *sme* stratum moleculare externum, *sg* stratum granulosum, *sp* stratum purkinjense

The IFA analysis of the index patient’s (P1) serum and CSF revealed a fine-granular IgG staining of the molecular layer on the rat hippocampus as well as on the rat and primate cerebella (Fig. 1, Additional file 1: Figure e-5). The hippocampal inner molecular layer showed a more intense staining than the outer molecular layer. Serum and CSF titers were 1:320 and 1:32, respectively, and the antibodies were of subclass IgG1, exclusively (see details in Table 1, Additional file 1: Figure e-5). Further monospecific analyses revealed no specific reactivity applying 30 different neural autoantigens recombinantly expressed in HEK293 cells: Hu, Yo, Ri, CV2, SOX1, PNMA1, PNMA2, ITPR1, Homer 3, CARP VIII, ARHGAP26, ZIC4, DNER/Tr, GAD65, GAD67, amphiphysin, recoverin, GABA B receptor, glycine receptor, DPPX, glutamate receptors (types NMDA, AMPA, mGluR1, mGluR5), LGI1, CASPR2, AQP4 (M1 and M23), MOG, and ATP1A3.

**Table 1:** Summary of the clinical and paraclinical features of six patients with anti-flotillin-1/2

	Patient 1 (P1)	Patient 2 (P2)	Patient 3 (P3)	Patient 4 (P4)	Patient 5 (P5)	Patient 6 (P6)
Age (years) at diagnostic testing, gender	34, female	35, female	54, male	40, female	46, female	54, female
Disease duration at time of serum sampling (years)	0	3	0	18	0	8
Diagnosis	RRMS	RRMS	RRMS	RRMS	RRMS	RRMS
ON	Unilateral	No	Unilateral	Bilateral	P100 difference in the VEP	Unilateral
Imaging	Several brain lesions, no spinal lesion	Contrast-enhancing spinal lesion, no brain lesion	One spinal lesion and further brain lesions	Multiple white matter lesions including the temporal lobe, cerebellum, and spinal cord	Several subcortical T2 lesions, one DWI-positive ponto-medullar lesion, and 3 further spinal lesions	Contrast-enhancing lesions at atlanto-occipital level
McDonald’s criteria fulfilled	DIS, DIT, positive CSF	Relapses, positive CSF	Relapses, DIS, DIT, positive CSF	Relapses, DIS, DIT, positive CSF	DIS, DIT, positive CSF	Relapses, DIS, positive CSF
Expanded Disability Status Scale	2.0	2.0	2.0	3.5	2.0	2.0
Serum titer (tissue pattern on cerebellum/anti-flotillin-1/2)	1:320/1:3200	1:100/1:1000	1:100/1:1000	1:1000/1:10,000	1:1000/1:10,000	1:100/1:1000
OCB in CSF only	Yes	Yes	Yes	Yes	Yes	Yes
White cell count in CSF (cells/μl)	9	5	7	n.a.	6	2
CSF titer (tissue pattern on cerebellum/anti-flotillin-1/2)	1:32/1:100	Neg./1:3.2	1:10/1:100	n.a.	1:100/1:1000	n.a.
Intrathecal anti-flotillin-1/2 synthesis	Yes	No	Yes	n.a.^b^	Yes	n.a.^b^

*Flot-1/2* HEK293-flotillin-1/2, *RRMS* relapsing-remitting multiple sclerosis, *n.a.* not available, *DIS* dissemination over space, *DIS* dissemination over time

^b^CSF not available for anti-flotillin-1/2 testing

### Identification of flotillin as the target autoantigen

**Fig. 2:**
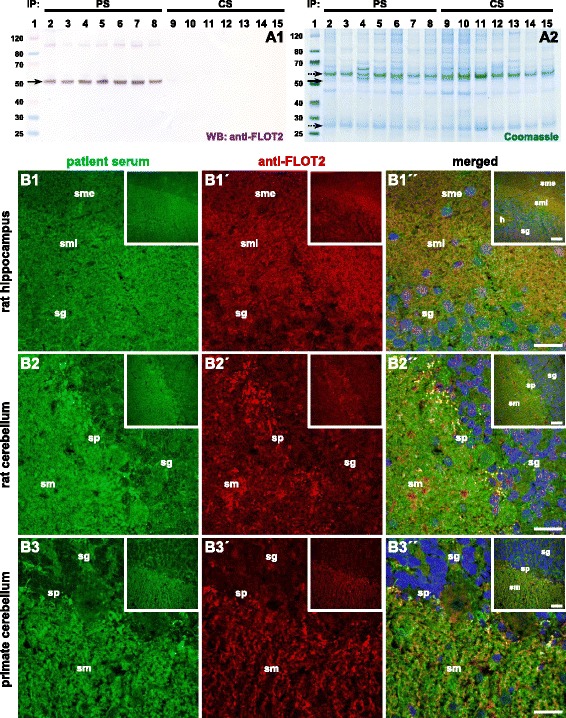
Histo-immunoprecipitation and antigen identification. Cryosections of rat or pig cerebellum were incubated with the serum (1:100), washed in PBS, and solubilized using detergents. The solution was incubated with protein-G-coated magnetic beads. The immunocomplexes were eluted by SDS and subjected to SDS-PAGE analysis and Western blot. **A** Western blot after incubation with anti-flotillin-2 (*A1*) and enzymatic visualization of antibody binding. Staining of SDS polyacrylamide gel with colloidal Coomassie (*A2*). Lane 1: molecular mass (kDa) marker; lanes 2–8: histo-immunoprecipitates of patient sera from rat cerebellum; lanes 9–15: histo-immunoprecipitates of control samples. The *arrow* indicates the position of the immunoprecipitated antigen 50 kDa while *dotted arrows* indicate the position of IgG heavy and light chain at 52 and 27 kDa, respectively. *PS* patient sample, *CS* control sample. **B** Immunofluorescence staining of rat hippocampus (*B1*) and cerebellum (*B2*) and primate cerebellum (*B3*) tissue sections with patient serum (*green*, *1–3*) and anti-flotillin-2 antibody (*red*, *1′–3′*). The merged images show localization of the reactivity in the same region including the more intense staining of the sm internum on the hippocampus (*1″–3″*). *Scale bar*: 50 μm (large images), 100 μm (inserts). *PS* patient serum, *CS* control serum, *h* hilus, *sg* stratum granulosum, *sm* stratum moleculare, *smi* stratum moleculare internum, *sme* stratum moleculare externum, *sp* stratum purkinjense

Immunocomplexes precipitated with the patient’s serum from the rat cerebellum contained proteins of approximately 50 kDa, as detected by SDS-PAGE, which were absent in equally prepared controls (Fig. 2A). Using MALDI-TOF, the 50-kDa proteins were identified as flotillin-1 and flotillin-2 from *Rattus norvegicus* (UNIPROT acc. # Q9Z1E1, Q9Z2S9) and *Sus scrofa* (#Q767L6, I3LFS8), respectively.

Western blot analysis of the immunoprecipitates exhibited a strong reaction at 50 kDa applying polyclonal rabbit anti-flotillin-1 and anti-flotillin-2 antibodies, respectively (Fig. 2A and Additional file 1: Figure e-1A). When used in IFA, the anti-flotillin-1 and anti-flotillin-2 antibodies produced fluorescence patterns on rat hippocampus as well as on rat and primate cerebella comparable to those generated by the patient samples (Fig. 2B and Additional file 1: Figure e-1B).

**Fig. 3:**
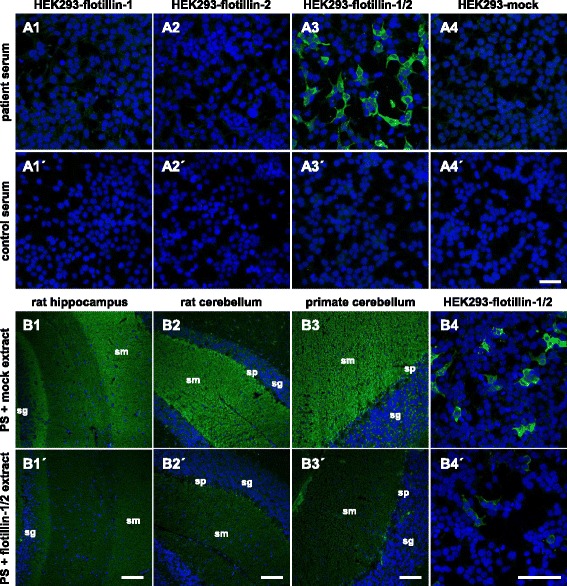
Immunofluorescence staining of recombinant flotillin and the neutralization of antibody reaction on tissue. **A** Immunofluorescence analysis of transfected HEK293 cells. Acetone-fixed recombinant HEK293 cells expressing flotillin-1 (*1*), flotillin-2 (*2*), flotillin-1 and flotillin-2 (*3*) or a mock-transfected control (*4*) were incubated with patient serum (*1–4*) or with control serum (*1′–4′*) (both 1:100). Cell nuclei were counterstained with TO-PRO-3 iodide (*blue*). Only HEK293-flotillin-1/2 cells reacted with the samples (*green*). *Scale bar*: 50 μm. **B** Neutralization of immunofluorescence reaction on neuronal tissues. Serum was pre-incubated with extracts of HEK293 cells transfected with empty control vector (*1–4*) or with the plasmid harboring flotillin-1/2 cDNA (*1′–4′*). The extract containing flotillin-1/2 greatly reduced or abolished the immune reaction of the serum on rat hippocampus (*1′*) and cerebellum (*2′*), primate cerebellum (*3′*), and HEK293-flotillin-1/2 (*4′*). The control extracts had no effect (*1–4*). Nuclei were counterstained by incubation with TO-PRO-3 iodide (*blue*). *Scale bar*: 50 μm. *PS* patient serum, *CS* control serum, *sg* stratum granulosum, *sm* stratum moleculare, *sp* stratum purkinjense

As a proof of correct antigen identification, the patient’s samples were then tested by IFA using recombinant HEK293 cells which expressed either flotillin-1, flotillin-2, or both proteins (Fig. 3A). Serum (Additional file 1: Figure e-3) and CSF (Additional file 1: Figure e-5) reacted with the cells co-expressing flotillin-1 and flotillin-2 after acetone fixation and in live-cell IFA (Additional file 1: Figure e-4) but not with those expressing individual flotillin-1 or flotillin-2. Endpoint titers were higher with the recombinant substrates than titers determined on tissue. Mock transfection did not result in any antibody binding.

The congruence of the autoantibody target and flotillin-1/2 was further demonstrated by the proof of a dose-dependent neutralization of antibody binding to brain tissue: An incubation of the patient’s samples with HEK293 lysate containing co-expressed flotillin-1 and flotillin-2 abolished the positive IFT reaction which did not happen if separately expressed flotillin-1 or flotillin-2 or lysates from mock-transfected HEK293 were added to the patient’s samples (Fig. 3B).

### Surface localization of autoantibody-binding recombinant flotillin-1/2

To study the membrane association of flotillins, acylation-deficient mutants were expressed in HEK293 cells. For this purpose, Cys5 and Cys17 of flotillin-1 and Cys4, Cys19, and Cys20 of flotillin-2 were replaced by serine [A: flotillin-1 wild-type + flotillin-2 wild-type; B: flotillin-1 wild-type + flotillin-2 (C4S, C19S, C20S); C: flotillin-1 (C5S, C17S) + flotillin-2 wild-type; D: flotillin-1 (C5S, C17S) + flotillin-2 (C4S, C19S, C20S)] (Additional file 1: Figure e-2). Using non-permeabilized HEK293 cells in a live cell-based assay, the patient serum and the antibodies against flotillin-1 and flotillin-2 only displayed reactions with the wild-type flotillin-1/2. Co-expression of acylation-deficient flotillin-1 (C5S, C17S) and flotillin-2 (C4S, C19S, C20S) impaired antibody binding to the flotillins and abolished the reactivity of patient serum and polyclonal antibodies, indicating that flotillins are not trafficked to the cellular surface without the attachment of fatty acids (Additional file 1: Figure e-4).

### Estimation of anti-flotillin-1/2 autoantibody prevalence

Anti-flotillin-1/2 status was determined in 521 samples referred for anti-AQP4 testing due to suspected autoimmune inflammatory CNS disorder (1) which had been found to be anti-AQP4-negative; (2) for which a general broad neural autoantibody screening, including the aforementioned parameters, had been conducted in the Clinical Immunological Laboratory, Lübeck (Germany); and (3) for which a neural tissue-reactive IgG autoantibody without known antigen specificity had been reported. Serum anti-flotillin-1/2 was detected in nine patients with titers of up to 1:10,000. For five of these patients, medical records were available (P2–P6 in Table 1). Patients P2, P3, and P5 also showed anti-flotillin-1/2 in CSF (titers 1:3.2, 1:100, and 1:1000). For P4 and P6, CSF was not available. Additional follow-up sera of the patients were analyzed when available and showed that the anti-flotillin-1/2 titers of P1, P2, and P4 were unaltered over a period of 18, 24, and 72 months, respectively, despite initiation of disease-modifying treatment for MS (interferon-β or natalizumab). The serum of P5 showed a reduction from 1:1000 to 1:320 7 weeks after plasma exchange, to which the patient had responded well clinically.

Screening of 538 serum samples from pre-diagnosed MS patients revealed anti-flotillin-1/2 autoantibodies in eight further patients (Additional file 1: Table e-2). All eight patient samples did not contain antibodies against AQP4 and MOG; however, screening of the remaining 530 samples uncovered 3 anti-AQP4- and 10 anti-MOG-positive sera in patients diagnosed with MS. Sera from 67 patients with various neural autoantibodies, including 38 with anti-AQP4 (titers up to 1:3200), and from 444 healthy participants (blood donors of both sexes, 18–67 years) were analyzed as specificity controls. None of the sera reacted with HEK293-flotillin-1, HEK293-flotillin-2, and HEK293-flotillin-1/2.

### Patient characteristics

In summary, 13 of the 14 anti-flotillin-1/2-positive patients for whom medical records could be evaluated had relapses, radiological signs of disseminated demyelination, and no evidence for a disorder other than MS, *ex post* fulfilling the revised diagnostic criteria of relapsing-remitting MS [24]. Providing direct evidence for the inflammatory nature of the disease, CSF analysis had revealed mild pleocytosis and/or CSF-specific oligoclonal band (OCB) in all patients. One patient had been diagnosed with secondary progressive MS. Thirteen (93%) patients were female. Nine (64%) patients had a history of optic neuritis. None of the patients displayed anti-AQP4 or anti-MOG antibodies. Where available (P1, P2, P3, and P5), CSF was found to harbor anti-flotillin-1/2. Specific antibody indices >4 indicative for intrathecal anti-flotillin-1/2 synthesis, as suggested by Reiber et al. for IFA analysis [25], were calculated for P1, P3, and P5. See Table 1 and Additional file 1 for more details.

## Discussion

We report on IgG autoantibodies against the flotillin-1/2 complex. The autoantibodies were revealed during the serological work-up of patients referred for anti-AQP4 testing as an unexpected finding. The patients’ sera and CSF reacted with cryosections of neural tissue but not with any of 30 previously established brain autoantigens in fixed recombinant cell-based IFA, including AQP4 and MOG. Histo-immunoprecipitation combined with mass spectrometry and recombinant expression identified the heterocomplex as the target antigen. All six index patients for whom medical records could be evaluated presented with a history of encephalomyelitis and/or inflammation of the optic nerve(s). *Ex post*, all patients fulfilled the revised diagnostic criteria for MS which prompted the search for anti-flotillin-1/2 in a cohort of 538 pre-diagnosed MS patients that revealed another 8 patients bearing the autoantibodies. Importantly, the antibodies were not detected in >500 controls.

Flotillin-1 and Flotillin-2 (synonyms: reggie-2 and reggie-1) were originally described to be upregulated in regenerating axons of goldfish retinal ganglion cells after traumatic injury of the optic nerve [22]. They are evolutionary well conserved in many eukaryotes including mammals [26, 27]. Human and rat flotillin-1 share 98% identity whereas the homology of human and rat/pig flotillin-2 is even 99%. In vertebrates, both proteins are ubiquitously expressed and abundant in striated muscle, adipose, lung tissue, and brain [15, 28]. On the cellular level, flotillins have been reported to be located in lipid rafts [28]. Functionally, flotillins have been shown to be involved in endocytosis, cell signaling, clustering of the amyloid precursor protein (APP) and amyloidogenic processing in neurons. Expression in glial cells has not been reported thus far. Moreover, artificial induction of flotillin expression in retinal ganglion cells in vivo leads to regeneration of subsequently crushed optic nerves in rats, thereby overcoming factors that inhibit this process in mammals under wild-type circumstances [27]. SiRNA-mediated downregulation of flotillin-2 affects neuronal differentiation and process formation in hippocampal neurons [29].

Despite of the abundant data on the individual flotillin proteins, information on expression, distribution, and function of flotillin-1 and flotillin-2 homo- and heterocomplexes is limited. Our own data indicate the presence of recombinant wild-type flotillin-1/2 on the surface of HEK293 cells whereas acylation-deficient but autoantibody-binding flotillin-1/2 is retained within the cells. Given that authentic flotillin-1/2 heterocomplexes were temporarily present on cell surfaces of neural cells in vivo or on signal-transducing exosomes [30], the autoantibodies could bear pathogenic potential. Furthermore, the antibodies univocally belong to the complement-activating IgG1 subclass, seem to be produced within the CNS, and remained detectable over the entire disease course in all three patients with long-term follow-up, and P5 favorably responded to plasma exchange. These facts are compatible with a role of anti-flotillin-1/2 in the inflammatory process. However, an involvement of anti-flotillin-1/2 and/or the flotillin proteins in MS pathogenesis is only speculative based on our data. The antibodies may as well be a result of an overshooting immune response during the inflammatory process and represent an immunological epiphenomenon.

In the six anti-flotillin-1/2-positive patients in whom the antibody was found prospectively and for whom we could evaluate medical records, testing for anti-AQP4 and anti-MOG was initially requested based on clinically assessed NMOSD core characteristics like myelitis and/or optic neuritis [10]. However, none met the latest international consensus criteria for NMOSD and all demonstrated “red flag” criteria according to that consensus, strongly suggesting another form of encephalomyelitis [10]. Neuroimaging; laboratory features, including the presence of typical MS lesions on brain MRI and of CSF-restricted oligoclonal bands; and fulfillment of the revised diagnostic criteria indicated MS in all patients [24]. Based on our prospective approach regarding the disease phenotype and the absence of anti-flotillin-1/2 in the controls, as well as based on the retrospective confirmation in a large cohort of pre-diagnosed MS patients, the flotillin-1/2 heterocomplex seems to be a B cell autoantigen in a subset of about 1–2% of MS patients.

## Conclusions

Taken together, the compiled data indicate flotillin-1/2 heterocomplex as a target antigen of autoantibodies in a subset of patients with *bona fide* MS. More precise data regarding the time point at which anti-flotillin-1/2 is induced, and the phenotype relation and the prevalence have to be assembled in large cohorts of patients with inflammatory demyelinating CNS disorders including CIS; acute disseminated encephalomyelitis; NMOSD, each with and without ON; and isolated ON in order to discern the diagnostic, therapeutic, and prognostic value of these autoantibodies. Furthermore, these future data will help to elucidate the pathological relevance and functional relationship of flotillin antibodies.

## Additional file

Additional file 1: Novel antibody against flotillin. (ZIP 138728 kb).

## Abbreviations

AQP4: Aquaporin-4
CIS: Clinically isolated syndrome
CSF: Cerebrospinal fluid
FITC: Fluorescein isothiocyanate
IFA: Indirect immunofluorescence assay
IP: Immunoprecipitation
MALDI-TOF: Matrix-assisted laser desorption/ionization-time of flight mass spectrometry
MRI: Magnetic resonance imaging
MS: Multiple sclerosis
OCB: Oligoclonal bands
ON: Optic neuritis
PAGE: Polyacrylamide gel electrophoresis
