# Supplementary material for: Identification of the flotillin-1/2 heterocomplex as a target of autoantibodies in bona fide multiple sclerosis
Source: J Neuroinflammation. 2017 Jun 23;14:123. doi: 10.1186/s12974-017-0900-z (PMC5481867; doi:10.1186/s12974-017-0900-z)
Supplement: Additional file 1: — Novel antibody against flotillin. (ZIP 138728 kb). [file 12974_2017_900_MOESM1_ESM.zip › 170518 Supplement Novel antibody against flotillin_final.docx]

**Abbreviations:** MALDI-TOF, Matrix-Assisted Laser Desorption/Ionization-Time Of Flight mass spectrometry; PAGE, polyacrylamide gel electrophoresis

###### Supplemental Materials and Methods

*SDS-PAGE and Western blot*

Proteins were analyzed by SDS-PAGE using the NuPAGE system (ThermoFisher Scientific, Dreieich, Germany). Separated proteins were either identified by mass spectrometric analysis or electrotransferred onto a nitrocellulose membrane by tank blotting with transfer buffer (ThermoFisher Scientific, Dreieich, Germany) according to the manufacturer’s instructions. The membranes were blocked with Universal Blot Buffer plus (Euroimmun, Lübeck, Germany) for 15 min and incubated with human serum or the polyclonal rabbit antibodies against flotillin-1 and flotillin-2 in Universal Blot Buffer plus for 3 hours, followed by 3 washing steps with Universal Blot Buffer (Euroimmun, Lübeck, Germany), a second incubation for 30 min with anti-human IgG-AP (Euroimmun, Lübeck, Germany) or anti-rabbit IgG-AP (Sigma-Aldrich, Heidelberg, Germany), 3 washing steps, and staining with NBT/BCIP substrate (Euroimmun, Lübeck, Germany).

1. *Mass spectrometry*
2. Mass spectrometry sample preparation was performed as reported by Koy *et al.* [1] Unless otherwise indicated, hardware, software, MALDI targets, peptide standards and matrix reagents were obtained from Bruker Daltonics (Bremen, Germany).
3. Briefly, samples were reduced with dithiothreitol and carbamido-methylated with iodoacetamide prior to SDS-PAGE. Proteins were visualized with Coomassie Brilliant Blue G-250 and visible protein bands were excised and discolored. After tryptic digest peptides were extracted and spotted with α-cyano-4-hydroxycinnamic acid onto a MTP AnchorChip™ 384 TF target.
4. MALDI-TOF/TOF measurements were performed with an Autoflex III smartbeam TOF/TOF200 System using flexControl 3.0 software. MS spectra for peptide mass fingerprinting (PMF) were recorded in positive ion reflector mode with 500 shots and in a mass range from 700 Da to 4000 Da. Spectra were calibrated externally with the commercially available Peptide Calibration Standard II and processed with flexAnalysis 3.0, and peak lists were analyzed with BioTools 3.2.
5. The Mascot search engine Mascot Server 2.3 (Matrix Science, London, UK) was used for protein identification by searching against the NCBI database limited to Mammalia. Search parameters were as follows: Mass tolerance was set to 80 ppm, one missed cleavage site was accepted, and carbamidomethylation of cysteine residues as well as oxidation of methionine residues were set as fixed and variable modifications, respectively. To evaluate the protein hits, a significance threshold of p<0.05 was chosen. For further confirmation of the PMF hits two peptides of each identified protein were selected for MS/MS measurements using the WARP feedback mechanism of BioTools. Parent and fragment masses were recorded with 400 and 1000 shots, respectively. Spectra were processed and analyzed as described above with a fragment mass tolerance of 0.7 Da.
6. *Cloning and recombinant expression of flotillin-1 and flotillin-2 in HEK293 cells*
7. The coding DNAs for human flotillin-1 (UNIPROT acc. # O75955) and flotillin-2 (# Q14254) in authentic or mutated [flotillin-1(C5S, C17S) and flotillin-2(C4S, C19S, C20S)] form were obtained by PCR on commercially available cDNA (Imagenes, Nottingham, UK) with the primers and synthetic DNA (eurofins MWG, Ebersberg, Germany) given in **Table e-1**. The amplification products were digested with the appropriate restriction enzymes and ligated with pTriEx-1 (Merck). The subunits were expressed individually or in conjunction in the human cell line HEK293 after ExGen500-mediated transfection (ThermoFisher Scientific, Dreieich, Germany) according to the manufacturer’s instructions. In order to prepare substrates for IFA, HEK293 cells were seeded on sterile cover glasses, transfected, and allowed to express flotillin-1 and flotillin-2 either individually or in conjunction for 48 hours. Cover glasses were washed with PBS, fixed with either acetone or 1.8% (v/v) formalin for 10 minutes at room temperature, air-dried, cut into millimeter-sized biochips and used as substrates in IFA as described. For live-cell IFA with transfected HEK293 cells, they were seeded in Lab-Tek II Chamber Slides (ThermoFisher Scientific, Dreieich, Germany) and transfected identically. Alternatively, cells were transfected in standard T-flasks and harvested after 5 days. The cell suspension was centrifuged at 1,500 x g, 4 °C for 20 min and the resulting sediment was resuspended with 100 mmol/l tris-HCl pH 7.4, 150 mmol/l sodium chloride, 2.5 mmol/l EDTA, 0.5% (w/v) deoxycholate, 1% (w/v) Triton X-100 containing protease inhibitors. The suspensions were stored in aliquots at -80 °C until further use.

*Calculation of the antibody index*

Intrathecal synthesis of IgG to the flotillin-1/2 complex was determined by calculating the antibody index (AI): AI = Q_IgG[spec]_/Q_IgG[total]_ (with Q_IgG[total]_ < Q_lim_) and AI = Q_IgG[spec]_/Q_lim_ (with Q_IgG[total]_ > Q_lim_) with Q_IgG[spec]_ = IgG_spec[CSF]_/ IgG_spec[serum]_, and Q_IgG[total]_ = IgG_total[CSF]_/IgG_total[serum]_. The upper reference range of Q_IgG[total]_ and Q_lim_, were calculated according to Reiber’s formula.[2] AI values >4 were considered positive.[3]

###### Supplemental Results

*Characterization of the patients*

Patient 1

The female index patient (34 y, **P1**), healthy in other respects, initially presented with blurred vision, retrobulbar pain, and reduced intensity of red color vision of the right eye which started five days before presentation in April 2013. Except oral contraceptive medication her medical history was unremarkable. In her maternal grandmother MS had been suspected; her mother’s brother suffered from a non-specified mental handicap. Neurological clinical examination did not reveal pathological symptoms. Ophthalmological examination proved impaired vision (right eye: 20/30, left: 20/20). Visual evoked potentials (VEP) were delayed and amplitudes were reduced on the right side. Somatosensory evoked potentials (SSEP) from the left leg were slightly delayed and amplitudes reduced. Magnetic evoked potentials (MEP) were normal. Magnetic resonance imaging (MRI) revealed contrast enhancement of the right optic nerve as well as several small subcortical white matter lesions (without contrast enhancement), compatible with a demyelinating disease. No spinal cord lesions were found. Routine blood testing including CRP and vitamin B12 was normal except for a slight increase in angiotensin-converting enzyme (23.4 IU/l, normal range: 8.3 - 21.4). Unspecified anti-nuclear autoantibodies were present at a titer of 1:320, testing for rheumatoid factor, pANCA, AMA, ENA-, anti-AQP4, anti-phospholid-, anti-*Borrelia*-, and anti-*Treponema* antibodies was negative. CSF examination revealed mild pleocytosis (9 cells/µl), normal total protein (269 mg/l), local IgG synthesis (53%), and oligoclonal bands (OCB). The patient received intravenous methylprednisolone pulse therapy (1g/d for 3 days) and subsequently treatment with interferon beta (IFN-beta)-1b was initiated.

Five months later, MRI revealed a new contrast enhancing lesion that confirmed the diagnosis of MS. At that time, the visual symptoms had completely resolved (visual acuity in both eyes 20/20) and VEP normalized. At last follow-up (22 months after onset), the patient was stable with no new symptoms. A control lumbar puncture revealed a persistent autochthonous antibody production with OCB in the CSF as well as mild pleocytosis (5 cells/µl).

Patient 2

This female patient (35 y, **P2**) initially presented in 2010 with a painful hemi-hypesthesia distal of Th4, clumsy one-legged jumping and thermaesthesia on the back without disturbances of micturition or defecation and without paresis. MEP and SSEP indicated a central demyelinating process. Spinal cord magnetic resonance imaging (MRI) revealed a left anterio-laterally localized Gd-enhancing myelon lesion on dens level, a more distally located herniated vertebral disc on cervical spine level C6/C7, and additional non-contrast enhancing lesions in the spinal cord. Brain MRI was normal. Cerebrospinal fluid (CSF) examination demonstrated very mild pleocytosis (5 cells/µl) and CSF-restricted oligoclonal bands (OCB) but was otherwise unremarkable, including ACE and β2-microglobulin levels. Serum analysis showed low-titer ANA (1:80), but no ANCA, ENA-, dsDNA-, AQP4-, or MOG- antibodies. High-dose glucocorticoids (1 g/d IVMP) were administered for 3 days resulting in a rapid improvement and complete disappearance of the symptoms.

Neurological examination in 2012 revealed a mild persisting hemiparesis, combined with cloniform Achilles tendon reflex on the other side, and a small circumscript hypesthesia of the hand of the same side. CSF analysis demonstrated mild pleocytosis (9 cells/µl) and OCB. MRI revealed several new demyelinating lesions in spinal cord and brain. VEP were normal. Symptoms promptly resolved under high-dose IVMP (1g/d for 3 days). Relapsing-remitting MS was suspected and treatment with IFN-beta initiated, under which the patient presented 4 and 8 months later with a stable disease course.

Control cerebral MRI 12 months later revealed no new lesions and no contrast-enhancing lesions. Four months later, the patient complained about a mild weakness of the right arm and leg under exercise, which partially resolved after re-administration of high-dose IVMP (1g/d).

In February 2014, she complained about a mild monoparesis of the left leg. SSEP and MEP were unchanged. VEP were again normal. Due to flu-like symptoms and the aversion of the patient against injections, therapy was switched to dimethyl fumarate (2 x 240 mg/d) in August 2014.

Control MRIs in 2014 and 2015 revealed no new lesions except for a signal alteration in the ventral medulla oblongata without contrast-enhancement, the significance of which was uncertain due to local signal pulsation artefacts. At last follow-up in July 2015, a reduced visual acuity in the right eye (20/25) was noted. No further attacks have occurred in the meanwhile.

Patient 3

This male patient (54 y, **P3**), healthy in other respects, was initially presented in December 2014 with movement-dependent retrobulbar pain and visual loss in the left eye (20/63). Neurological examination was otherwise normal.

Lumbar puncture revealed a mild CSF pleocytosis (7 cells/µl), weakly positive OCB only in the CSF, a slightly elevated CSF/serum IgG ratio (4.3; normal range <4.2) and IgG index (0.8; normal range < 0.7). IgM antibody titers CSF against measles were marginally elevated. IgG antibody indices (AI) for varicella virus, measles, and rubella were elevated (anti-measles IgG AI 4.4 [normal range < 1.5]; anti-varicella IgG AI 1.7 [normal range < 1.5]; anti-rubella IgG AI 5.5 [normal range < 1.5]) as typically seen in patients with MS.

Brain MRI revealed contrast enhancement of the left optic nerve as well as several small subcortical T2-weighted, non-contrast enhancing white matter lesions, compatible with a demyelinating disease such as multiple sclerosis. Moreover, an additional, non-enhancing spinal cord lesion at C3 was detected. Routine blood testing was normal except for a slightly enhanced leucocyte count (14.18 cells/μl; normal range 3.91-12.68).

The patient was treated with IVMP pulse therapy (500 mg/d for 3 days) for suspected autoimmune optic neuritis, which was followed by rapid and complete remission. Therapy with pegylated IFN-beta-1a *s.c*. was started. At last follow-up 6 months later, no further attacks have occurred.

Patient 4

This female patient (40 y, **P4**) experienced her first myelitis episode in 1997 with hypesthesias in both legs. She was diagnosed with MS a few months later based on typical white matter lesions on brain and spinal MRI as well as presence of OCB in the CSF. After three relapses, one of which was associated with an epileptic seizure and ataxia, and because of an increase in lesion load on both cerebral as well as spinal MRI treatment with IFN-beta-1a (3 🞨 44 µg s.c./week) was started in early 2008. However, six months later she experienced another severe attack with paresis of the right leg and ataxia. Therefore, therapy was switched to natalizumab in July 2008 but stopped after four infusions due to increasing infusion related side effects (urticaria and dizziness). Natalizumab serum antibodies were negative at that time. Another severe attack with bilateral optic neuritis (ON) occurred four months later. As vision did not improve after two cycles of high dose glucocorticoid therapy, plasma exchange (PEX) was performed, which resulted in remission. Treatment with natalizumab was restarted in March 2009 with concomitant premedication including prednisolone 100 mg, dimetindene maleate and paracetamol. Natalizumab was now well tolerated and premedication could be tapered of after six months. At last follow-up in 2015, the patient was clinically stable with no attacks since 2009. Brain MRI showed multiple deep white matter lesions including the temporal lobe, hippocampus and cerebellum as well as multiple spinal cord lesions.

Patient 5

This previously healthy 46-year-old female (**P5**) presented with severe right occipital and nuchal headache (pain scale 10/10), sensory disturbances and severe nausea and vomiting. Symptoms had started 3 weeks ago, but were initially mild and present only intermittently. At admission, neurological examination revealed paresthesia, hypesthesia and hypalgesia of the right half of the face, hypesthesia of the right leg, unsteady gait with a strong tendency to fall, and a spontaneous nystagmus to the left (NIHSS 1; modified Rankin scale 4). While computed tomography (CT), CT angiography, MRI angiography and duplex ultrasonography were normal, T2-weighted MRI imaging of the brain showed single subcortical, hyperintense white matter lesions suggestive of multiple sclerosis. Diffusion-weighted MRI demonstrated in addition a ponto-medullary lesion, which was very small at admission but had significantly increased in size at follow-up MRI several days later. Spinal MRI revealed three oval lesions in the cervical spinal cord at C2 (16 mm), C3 (7 mm), and C7 (10 mm) and a single lesion in the thoracic spinal cord at Th3/4 (4 mm) without gadolinium enhancement. Lumbar puncture showed slight pleocytosis (6 cells/µl), CSF-restricted OCB, quantitative evidence for intrathecal IgG (intrathecal IgG fraction 36.87%) and IgA but not IgM synthesis and a mildly elevated CSF protein concentration; no blood CSF barrier dysfunction was noted. Blood examination (including CRP, leukocyte counts, ANA/ENA, dsDNA antibodies, lupus coagulant, ANCA, cardiolipid antibodies, rheumatoid factor, aquaporin-4 antibodies, *Borrelia burgdorferi*, *Treponema pallidum*, HIV) was normal except for mild hyponatremia (133 mmol/l) and low TSH serum level (0.52 mU/l; reference range 0.66-5.45). An X-ray of the chest did not reveal any evidence for sarcoidosis, tuberculosis, or lung cancer. Shortly after admission, the patient complained about visual disturbances and transient but severe dizziness. VEP were initially normal but showed a difference in latencies when repeated two weeks after admission. At that time-point, she transiently developed hypesthesia of the previously unaffected left leg and paraparesis; in addition, mild hypothermia was noted (34.9°C). A diagnosis of relapsing-remitting MS was made. Two cycles of IVMP (1g/d for 5 days) did not result in clinical improvement. By contrast, five subsequent plasma exchanges (PEX) were followed by substantial amelioration of the patient’s ataxia and nystagmus (beginning with the second PEX treatment). At discharge, she still suffered from hypesthesia of the right cheek, neck and tongue, difficulties reading, and a slightly unsteady gait. While the supraventricular white matter lesions were unchanged on a follow-up MRI performed around one month after admission, the medullary lesion was now detectable also on T2 imaging and contrast-enhanced T1 imaging but had decreased in size. A follow-up VEP showed improved latencies and amplitudes, although a slight difference in latencies between the right and the left eye was still present. A follow-up sample taken around 7 weeks after PEX was still positive for flotillin-1/2 antibodies, though at a much lower titer (1:320). Treatment with natalizumab was initiated at that time-point.

*Verification of flotillin expression in HEK293 cells*

Anti-flotillin-1 produced a bright cytoplasmic staining in IFA in acetone-fixed HEK293 cells that had been transfected with the expression constructs coding for flotillin-1 wild-type or flotillin-1(C5S, C17S) individually or in conjunction with the constructs for the flotillin-2 variants. Conversely, HEK293 cells transfected with the expression constructs coding for flotillin-2 wild-type or flotillin-2(C4S, C19S, C20S) were similarly stained by anti-flotillin-2. Analysis by Western blot showed a band at 50 kDa using anti-flotillin-1 when cells transfected with the expression constructs coding for flotillin-1 wild-type or flotillin-1(C5S, C17S) individually or in conjunction with the constructs for the flotillin-2 variants were analyzed (**Fig. e-2**). Conversely, anti-flotillin-2 produced a 50 kDa band when cells transfected with the expression constructs coding for flotillin-2 wild-type or flotillin-2(C4S, C19S, C20S) were analyzed (**Fig. e-2**).

**Table e-1. cDNA clones and primers**

Coding regions for the individual subunits of flotillin were prepared by PCR with the following primers and enzymatic digestion.

| **Subunit** | **Template designation** | **Cleavage site** | **Primers (5’->3’)** |
| --- | --- | --- | --- |
| FLOT1 | IRQMp5018E075D | BsmBI | ATA CGT CTC GCA TGT TTT TCA CTT GTG GCC |
|  |  | BsmBI | TAT CGT CTC CTC GAT CAG GCT GTT CTC AAA G |
| FLOT1 (C5S, C17S) | pTriEx-1-FLOT1 | BsmBI | ATA CGT CTC GCA TGT TTT TCA CTT CTG GCC CAA ATG AGG CGA TGG TGG TCT CCG GGT TCT CC |
|  |  | BsmBI | TAT CGT CTC CTC GAT CAG GCT GTT CTC AAA G |
| FLOT2 | synthetic construct | BsBI | gaa gac acc atg ggc aat tgc cac acg gtg ggc ccc aac gag gca ctg gtg gtc tca ggg ggc tgt tgt ggt tct gac tac aag cag tat gtg ttt ggc ggc tgg gct tgg gcc tgg tgg tgt atc tcg gac act cag agg att tcc cta gag att atg acg ttg cag ccc cgc tgt gag gac gta gag acg gcc gag ggg gta gct tta act gtg acg ggt gtc gcc cag gtg aag atc atg acg ggt ctt c |
|  | IRBPp993D0417D | BsBI | ATA GAA GAC TAT GAC GGA GAA GGA GCT CC |
|  |  | BsBI | TAT GAA GAC CTG CGG CGA TCT TGG CAG CAA TCT G |
|  |  | BsBI | ATA GAA GAC TGC CGC ACC CCT GAC TAA AGT C |
|  |  | BsBI | TAT GAA GAC CGT CGA TTA CAC CTG CAC ACC AGT GGC TTT CTT GAT C |
| FLOT2 (C4S, C19S, C20S) | pTriEx-1-FLOT2 | BsBI | ATA GAA GAC AGC ATG GGC AAT TCC CAC ACG GTG GGA CCC AAC GAG GCA CTG GTG GTC TCA GGG GGC TCT TCT GG |
|  |  | BsBI | TAT GAA GAC CGT CGA TTA CAC CTG CAC ACC AGT GGC TTT CTT GAT C |

1. **Table e-2. Summary of the clinical and paraclinical features of eight additional patients with anti-flotillin-1/2**
2. CSF was not available for anti-flotillin-1/2 testing. Flot-1/2 = HEK293-flotillin-1/2, RRMS = relapsing-remitting multiple sclerosis, SPMS = secondary progressive multiple sclerosis, Gd+ = Gadolinium enhancement, n.a. = not available, DIS = dissemination over space, DIS = dissemination over time. * based on OCB in CSF but not in serum. ** Clinical presentation at the time serum was taken, may have changed in the further clinical course. *** Data as close to the time point serum was taken as available.

|  | **665** | **679** | **765** | **861** | **1017** | **1036** | **Mü1** | **Mü2** |
| --- | --- | --- | --- | --- | --- | --- | --- | --- |
| Age (years) at diagnostic testing, gender | 40, female | 64, female | 60, female | 53, female | 58, female | 52, female | 55, female | 57, female |
| Disease duration at time of serum sampling (years) | 11 | 13 | 19 | 18 | 18 | 25 | 2 | 5 |
| Diagnosis (**) | RRMS | SPMS with superimposed relapses | RRMS | SPMS | RRMS | RRMS | RRMS | RRMS |
| ON | Classic clinical presentation of unilateral ON left | Marked prolonged P100-latency on both eyes of which patient is not aware | no | Prolonged P100-latency (132 ms OD, 128.4 ms OS), complains about smoldering onset of burry vision of both eyes | Several instances of uni- and bilaterial ON | Unilateral right | Two instances of unilateral ON | No |
| Imaging*** | Multiple non contrast-enhancing para- and periventricular white matter lesions, no Gd-enhancement. No spinal cord lesion | Multiple supra- and infratentorial white matter lesions.  No Gd+ administered.  No spinal cord imaging | Multiple white matter lesions subependymal and subcortical, no infratentorial lesion.  No Gd+-enhancing lesions.  No spinal cord imaging. | Massive -partly confluent- supra- and infratentorial white matter lesions. Brainstem involvement. No Gd+ enhancing lesions (09/2005). No spinal cord imaging. | Multiple white matter lesions subcortical, periventricular. One larger infratentorial lesion in the left crus cerebri | n.a. | Multiple non contrast-enhancing juxtacortical and periventricular white matter lesions, 1 thalamic grey matter lesion, 1 optic nerve lesion, no infratentorial or spinal cord lesions | Multiple non contrast-enhancing juxtacortical and periventricular white matter lesions, 1 contrast-enhancing hippocampal grey matter lesion, 1 spinal lesion, no optic nerve or infratentorial lesion |
| McDonald‘s criteria fulfilled (2010) | Relapses, DIS, DIT  positive CSF in 2008 | Relapses, DIS, DIT, positive CSF | Relapses | DIS, DIT, former relapses, positive CSF | DIT, DIS, relapses | Relapses | Relapses, DIS | Relapses, DIS, DIT, positive CSF |
| Expanded Disability Status Scale | 2.0 | 6.0 | 3.0 | 5.5 | 1.0 | 1.0 | 2.0 | 3.5 |
| Serum titer  (tissue pattern on cerebellum / anti-flotillin-1/2) | 1:32 / 1:1.000 | 1:320 / 1:3.200 | 1:32 / 1:1.000 | 1:32 / 1:100 | 1:32 / 1:32 | 1:32 / 1:320 | 1:32 / 1:100 | 1:10 / 1:320 |
| OCB in CSF only*** | Yes | Yes | n.a. | Yes | No | n.a. | No | Yes |
| White cell count in CSF (cells/µl)*** | 9 | 4 | n.a. | 12 | 24 | n.a. | 3 | 7 |

1. **Figure e-1. Histo-immunoprecipitation and antigen identification.**

Cryosections of rat cerebellum were incubated with the serum (1:100), washed in PBS and solubilized using detergents. The solution was incubated with protein-G-coated magnetic beads. The immunocomplexes were eluted by SDS and subjected to SDS-PAGE analysis and Western blot.

**A**: Western blot after incubation with anti-flotillin-1 (**A1**) and enzymatic visualization of antibody binding. Staining of SDS polyacrylamide gel with colloidal coomassie (**A2**). Lane 1: molecular mass (kDa) marker, lanes 2-8: histo-immunoprecipitates of patient sera from rat cerebellum, lanes 9-15: histo-immunoprecipitates of control samples. The arrow indicates the position of the immunoprecipitated antigen 50 kDa while dotted arrows indicate the position of IgG heavy and light chain at 52 kDa and 27 kDa, respectively. PS: patient sample; CS: control sample.

**B**: Immunofluorescence staining of rat hippocampus (**B1**) and cerebellum (**B2**) and primate (**B3**) cerebellum tissue sections with serum (green, **1-3**) and anti-flotillin-1 antibody (red, **1’-3’**). The merged images show localization of the reactivity in the same region including the more intense staining of the stratum moleculare internum hippocampus (**1’’-3’’**). Scale bar: 50 µm (large images), 100 µm (inserts).

1. **Figure e-2. Verification of flotillin expression in HEK293 cells**

Western blot after incubation with anti-flotillin-1 (**A**) or anti-flotillin-2 (**B**) and enzymatic visualization of antibody binding. Lane 1: molecular weight marker (kDa), lane 2: HEK-control, lane 3: HEK293-flotillin-1 wt, lane 4: HEK293-flotillin-2 wt, lane 5 : HEK293-flotillin-1 (C5S, C17S), lane 6 : HEK293-flotillin-2 (C4S, C19S, C20S), lane 7 : HEK293-flotillin-1 wt + flotillin-2 wt, lane 8 : HEK293-flotillin-1 wt + flotillin-2 (C4S, C19S, C20S), lane 9 : HEK293-flotillin-1 (C5S, C17S) + flotillin-2 (C4S, C19S, C20S), lane 10 : HEK293-flotillin-1 (C5S, C17S) + flotillin-2 wt. The arrows indicate the position of the recombinant flotillins at 50 kDa.

1. **Figure e-3. Double staining of recombinant HEK293 cells**
2. Immunofluorescence analysis of Acetone-fixed HEK293 cells expressing flotillin-1 (**1, 5**), flotillin-2 (**2, 6**), flotillin-1 & -2 (**3, 7**) or a mock-transfected control (**4, 8**) with patient serum and anti-flotillin-1 antibody (**1-4**) or with patient serum and anti-flotillin-2 antibody (**5-8**). Co-localization of the reactivity is exclusively visible on cells transfected with the co-expression of flotillin-1 and flotillin-2 (**3, 7**). Mock-transfected HEK293 cells do not show any reactivity. Scale bar: 50 µm.

**Figure e-4. Surface localization of recombinant flotillin-1/2**

IFA with **living** HEK293 cells expressing wild-type flotillin-1/2 (**A, D, G, J**) or flotillin-1(C5S, C17S) + flotillin-2(C4S, C19S, C20S) (**B, E, H, K**). Patient serum (**A-C**), anti-flotillin-1 antibody (**G-I**) and anti-flotillin-2 antibody (**J-L**) reacts exclusively with HEK293 cells transfected with wild-type flotillin-1/2 (**A, G, J**). Mock-transfected HEK293 cells (**C, F, I, L**) and control serum (**D-F**) do not display any reactivity. Scale bar: 50 µm.

**Figure e-5. Immunofluorescence analysis with patient liquor and subclass determination of patient autoantibody**

Cryosections of rat hippocampus (**A1**) and cerebellum (**A2**) as well as primate cerebellum (**A3**) were incubated with patient liquor (1:1) in the first step, and with Alexa Fluor 488 labeled goat anti-human IgG in the second step (green). Nuclei were counterstained by incubation with TO-PRO-3 iodide (blue). A fine-granular staining of the stratum moleculare (sm) was obtained. On hippocampus the sm internum was more intense than the sm externum similar to the signal caused by patient serum. Immunofluorescence staining of HEK293 cells expressing flotillin-1/2 with patient liquor (**B1**) and co-localization of patient liquor with anti-flotillin-1 antibody (B2) and anti-flotillin-2 antibody (**B3**).

Subclass determination of patient autoantibody by immunofluorescence analysis with specific secondary antibodies against IgG1, 2, 3, 4 and total IgG as control revealed reactivity of patient serum only when anti-IgG1- (**C1**) and anti-IgG- (**C2**) specific antibodies were used for detection.

Scale bar: 50 µm (large images), 100 µm (inserts).

Reference List

1. Koy C, Mikkat S, Raptakis E, Sutton C, Resch M, Tanaka K, and Glocker MO. Matrix-assisted laser desorption/ionization- quadrupole ion trap-time of flight mass spectrometry sequencing resolves structures of unidentified peptides obtained by in-gel tryptic digestion of haptoglobin derivatives from human plasma proteomes. Proteomics. 2003;3:851-858.

2. Reiber H. Cerebrospinal fluid--physiology, analysis and interpretation of protein patterns for diagnosis of neurological diseases. Mult Scler. 1998;4:99-107.

3. Reiber H, Lange P. Quantification of virus-specific antibodies in cerebrospinal fluid and serum: sensitive and specific detection of antibody synthesis in brain. Clin Chem. 1991;37:1153-1160.
