# Supplementary material for: Identification of the flotillin-1/2 heterocomplex as a target of autoantibodies in bona fide multiple sclerosis
Source: J Neuroinflammation. 2017 Jun 23;14:123. doi: 10.1186/s12974-017-0900-z (PMC5481867; doi:10.1186/s12974-017-0900-z)
Supplement: Additional file 1: — Novel antibody against flotillin. (ZIP 138728 kb). [file 12974_2017_900_MOESM1_ESM.zip › JNEU_Figure_e-1.pptx]

## Slide 1
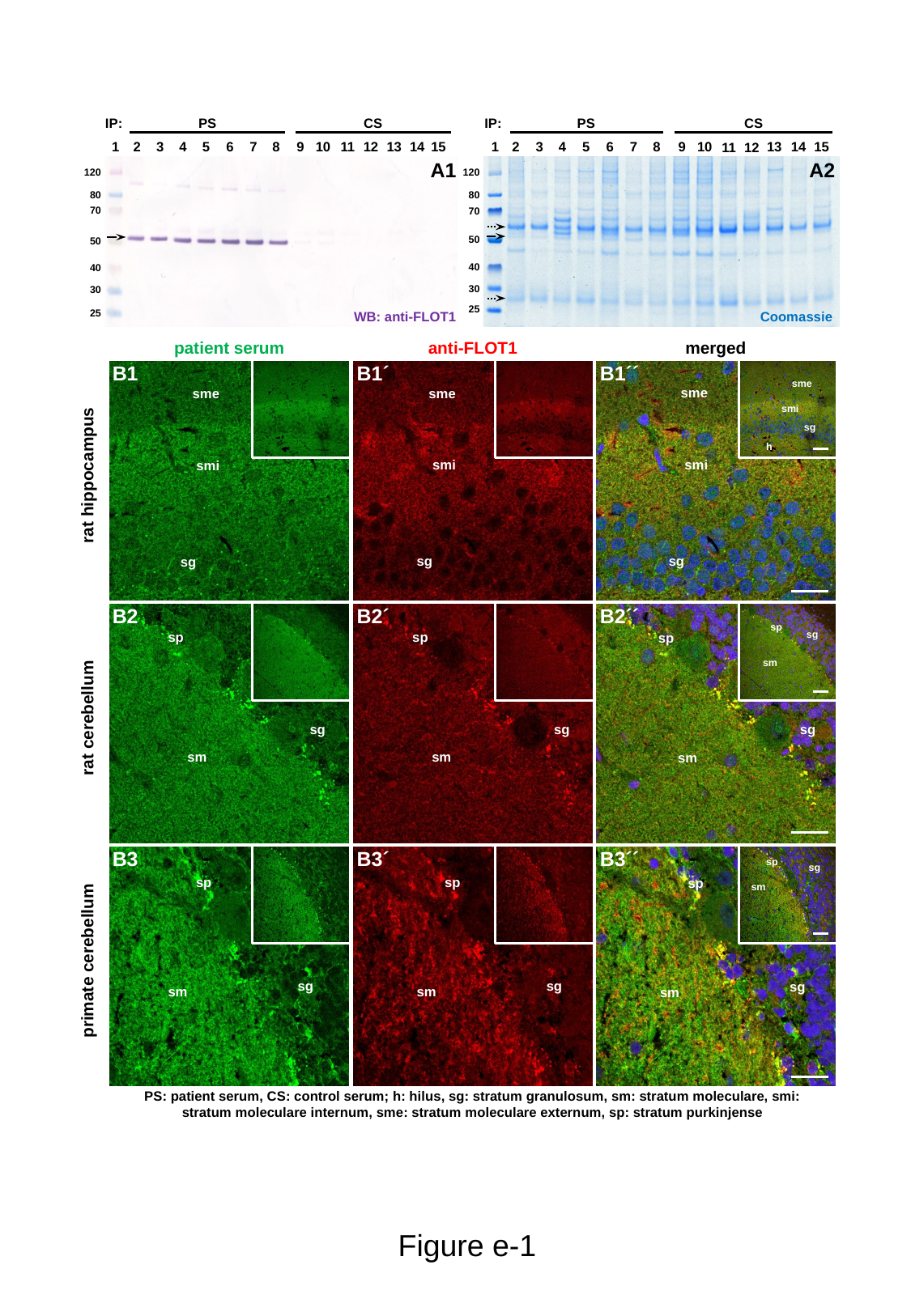

IP:
PS
CS
IP:
PS
CS
1
2
3
4
5
6
7
8
9
10
11
12
13
14
15
1
2
3
4
5
6
7
8
9
10
13
14
15
11
12
A1
A2
120
120
80
80
70
70
50
50
40
40
30
30
25
25
WB: anti-FLOT1
Coomassie
patient serum
anti-FLOT1
merged
B1
B1´
B1´´
sme
sme
sme
sme
smi
rat hippocampus
sg
h
smi
smi
smi
sg
sg
sg
B2
B2´
B2´´
sp
sg
sp
sp
sp
sm
rat cerebellum
sg
sg
sg
sm
sm
sm
B3
B3´
B3´´
sp
sg
sp
sp
sp
sm
primate cerebellum
sg
sg
sg
sm
sm
sm
PS: patient serum, CS: control serum; h: hilus, sg: stratum granulosum, sm: stratum moleculare, smi: stratum moleculare internum, sme: stratum moleculare externum, sp: stratum purkinjense
Figure e-1
