# Supplementary material for: Identification of the flotillin-1/2 heterocomplex as a target of autoantibodies in bona fide multiple sclerosis
Source: J Neuroinflammation. 2017 Jun 23;14:123. doi: 10.1186/s12974-017-0900-z (PMC5481867; doi:10.1186/s12974-017-0900-z)
Supplement: Additional file 1: — Novel antibody against flotillin. (ZIP 138728 kb). [file 12974_2017_900_MOESM1_ESM.zip › JNEU_Figure_e-2.pptx]

## Slide 1
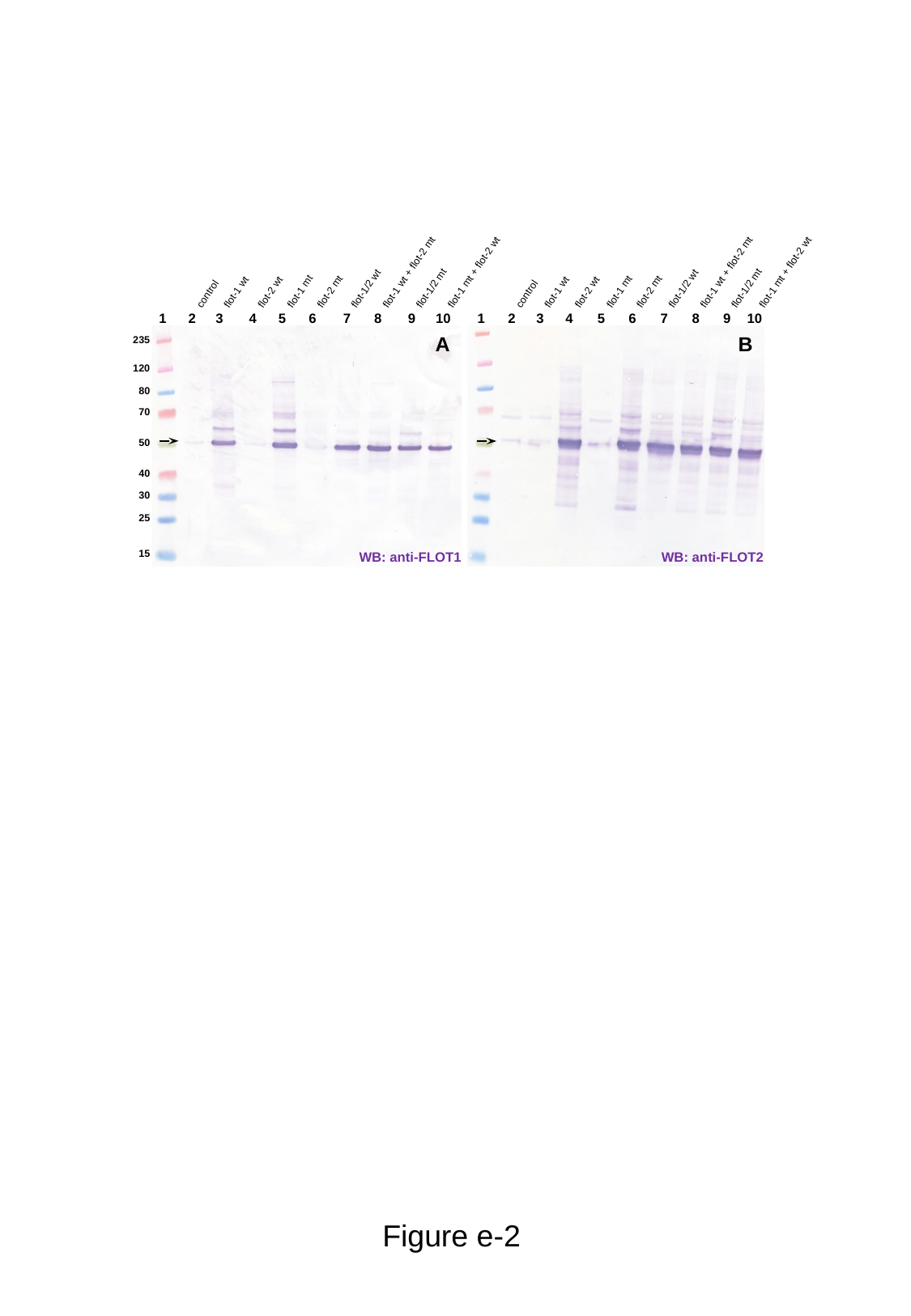

flot-1 wt + flot-2 mt
flot-1 mt + flot-2 wt
flot-1 wt + flot-2 mt
flot-1 mt + flot-2 wt
flot-1/2 mt
flot-1/2 mt
flot-1/2 wt
flot-1/2 wt
flot-1 mt
flot-2 mt
flot-1 mt
flot-2 mt
flot-1 wt
flot-2 wt
flot-1 wt
flot-2 wt
control
control
1
2
3
4
5
6
7
8
9
10
1
2
3
4
5
6
7
8
9
10
A
B
235
120
80
70
50
40
30
25
15
WB: anti-FLOT1
WB: anti-FLOT2
Figure e-2
