# Supplementary material for: Identification of the flotillin-1/2 heterocomplex as a target of autoantibodies in bona fide multiple sclerosis
Source: J Neuroinflammation. 2017 Jun 23;14:123. doi: 10.1186/s12974-017-0900-z (PMC5481867; doi:10.1186/s12974-017-0900-z)
Supplement: Additional file 1: — Novel antibody against flotillin. (ZIP 138728 kb). [file 12974_2017_900_MOESM1_ESM.zip › JNEU_Figure_e-4.pptx]

## Slide 1
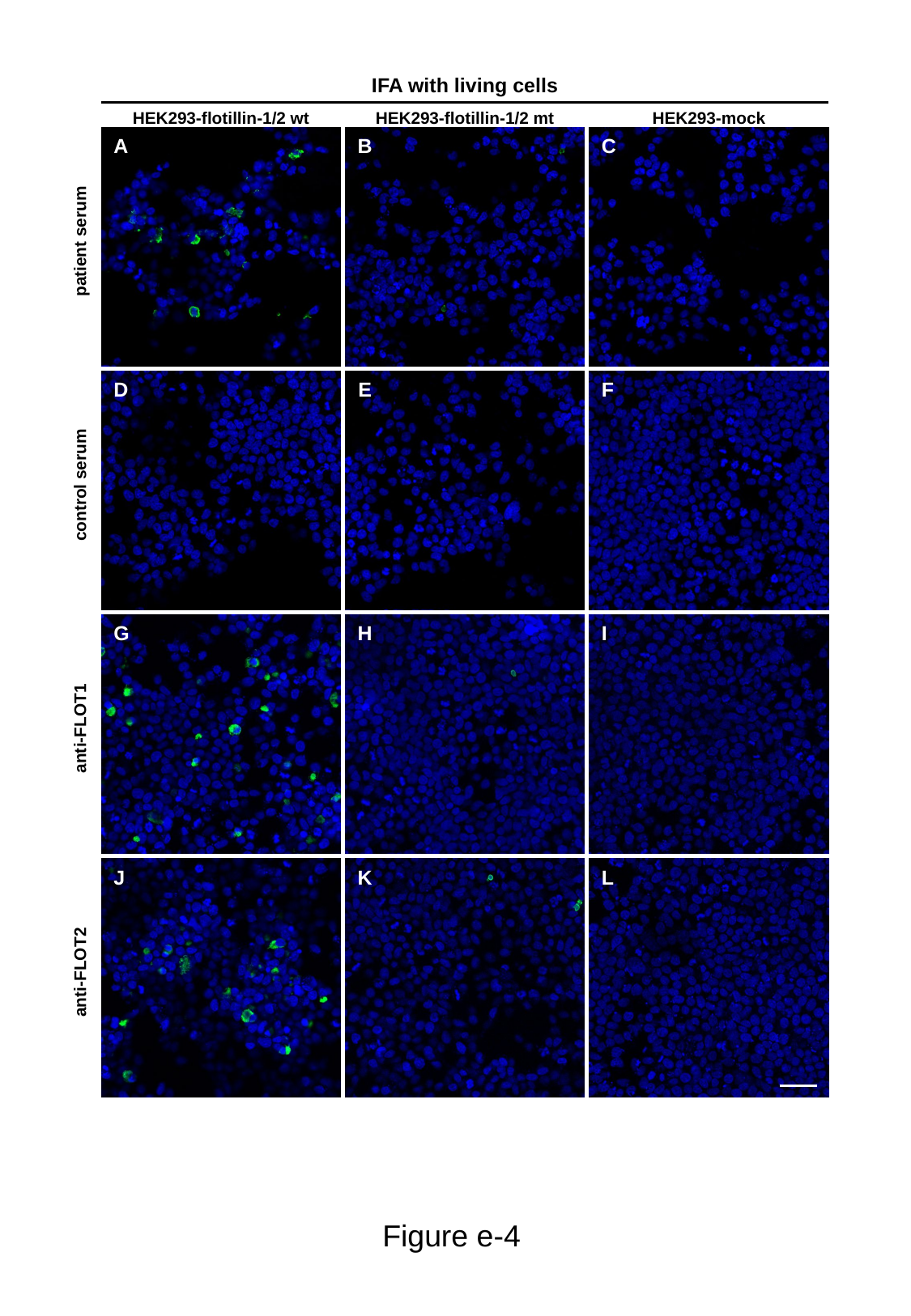

IFA with living cells
HEK293-flotillin-1/2 wt
HEK293-flotillin-1/2 mt
HEK293-mock
A
B
C
patient serum
D
E
F
control serum
G
H
I
anti-FLOT1
J
K
L
anti-FLOT2
Figure e-4
