# Supplementary material for: Identification of the flotillin-1/2 heterocomplex as a target of autoantibodies in bona fide multiple sclerosis
Source: J Neuroinflammation. 2017 Jun 23;14:123. doi: 10.1186/s12974-017-0900-z (PMC5481867; doi:10.1186/s12974-017-0900-z)
Supplement: Additional file 1: — Novel antibody against flotillin. (ZIP 138728 kb). [file 12974_2017_900_MOESM1_ESM.zip › JNEU_Figure_e-5.pptx]

## Slide 1
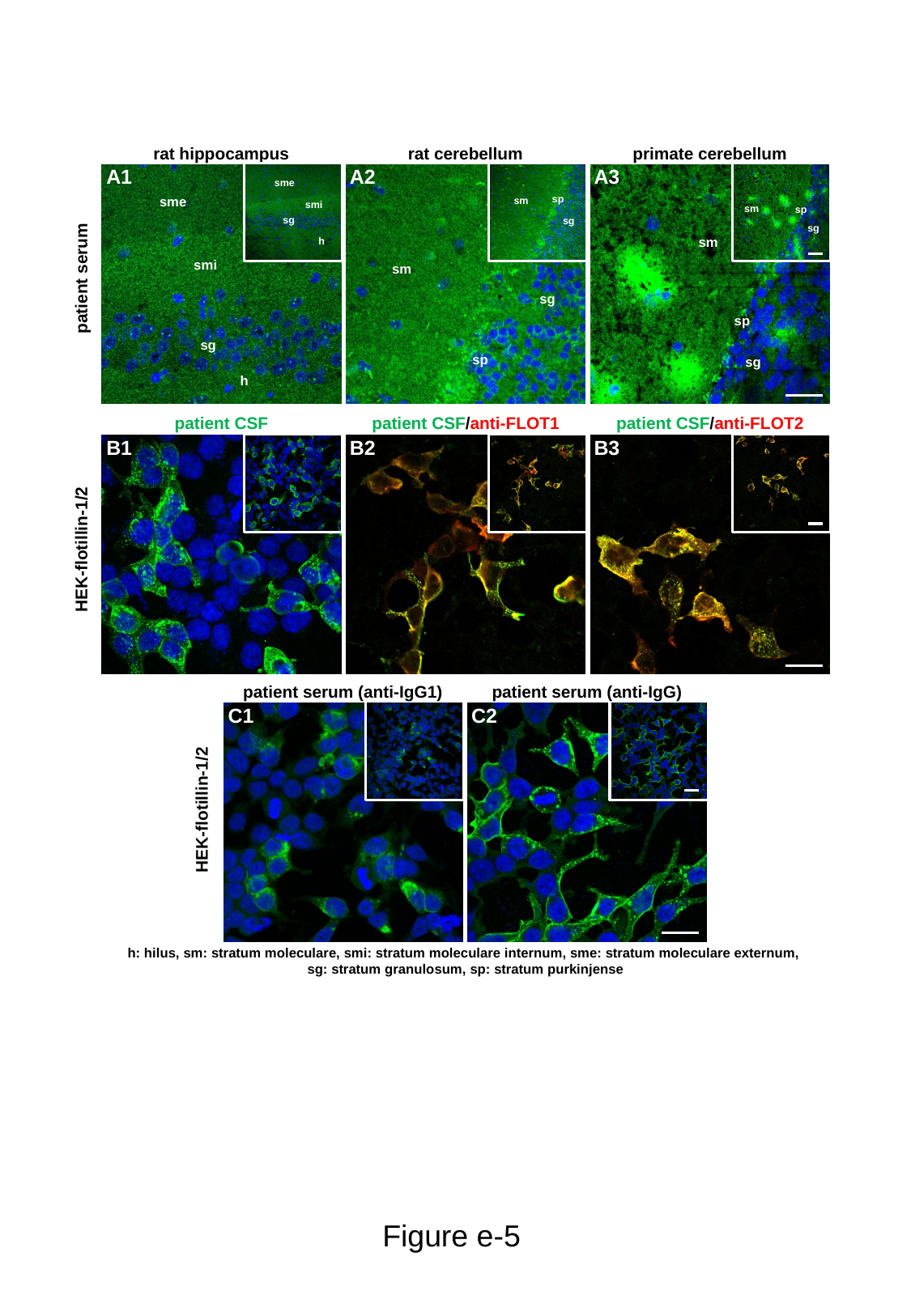

rat hippocampus
rat cerebellum
primate cerebellum
A1
A2
A3
sme
sp
sme
sm
smi
sm
sp
sg
sg
sg
patient serum
sm
h
smi
sm
sg
sp
sg
sp
sg
h
patient CSF
patient CSF/anti-FLOT1
patient CSF/anti-FLOT2
B1
B2
B3
HEK-flotillin-1/2
patient serum (anti-IgG1)
patient serum (anti-IgG)
C1
C2
HEK-flotillin-1/2
h: hilus, sm: stratum moleculare, smi: stratum moleculare internum, sme: stratum moleculare externum,
sg: stratum granulosum, sp: stratum purkinjense
Figure e-5
